# Pain Associated With Monkeypox Virus: A Rapid Review

**DOI:** 10.7759/cureus.34697

**Published:** 2023-02-06

**Authors:** Alejandro Hallo-Carrasco, Christine L Hunt, Christian C Prusinski, Jason S Eldrige, Kimberly H McVeigh, Mark Friedrich B Hurdle, Larry J Prokop, Sahil Gupta

**Affiliations:** 1 Pain Medicine, Mayo Clinic, Jacksonville, USA; 2 Library Services, Mayo Clinic, Rochester, USA

**Keywords:** acute pain management, acute pain, monkeypox complications, monkeypox treatment, monkeypox virus

## Abstract

International concerns for another pandemic arose after emerging reports of an ongoing outbreak of the monkeypox virus (MPXV) in Europe and the United States in 2022. Severe pain is one of the most distressing complications for patients in the current outbreak, but there is a general paucity of relevant peer-reviewed medical literature from which to draw clear recommendations on appropriate pain therapies. The Centers for Disease Control recently published a letter in July 2022 urging providers to conduct further studies concerning pain management. Thus, a rapid literature search was conducted in accordance with Preferred Reporting Items for Systematic Reviews and Meta-Analyses (PRISMA) guidelines. A comprehensive search of several databases from inception until August 19, 2022, was conducted. All published studies describing pain in patients who tested positive for MPXV with original data and written in English were included. Sixty-nine studies were initially identified for screening. After initial screening, 27 papers were considered for full-text review, and 15 papers met the inclusion criteria. A total of 1043 positive cases were included in this study. Most patients were men. Treatment options proposed by the authors include acetaminophen, ibuprofen, opioids, lidocaine gel, metamizole, and rectal suppositories containing emollients or steroids with oral laxatives for severe anal pain. Although most cases were mild requiring outpatient treatment, a considerable number of patients were admitted due to serious complications. Severe pain was often the reason to seek medical attention and hospital admission for pain control. Analgesic plans included oral and topical analgesia. In severe cases, pain was managed with opioids. To our knowledge, this rapid review is the first study to comprehensively summarize proposed treatments for pain associated with MPXV. Guidelines may be needed to help direct the best management to avoid morbidity in patients, particularly as adjuvants may play a key role but are not commonly utilized in published reports.

## Introduction and background

International concerns for another pandemic arose in early 2022 after countries reported an ongoing outbreak of the monkeypox virus (MPXV) in Europe and the United States [1]. The rising rate of community transmission and varying clinical presentations have concerned the medical community: 15,000 US cases and 41,000 global cases of monkeypox have been reported outside of historically endemic areas in more than 70 countries over the last several months. This surge in incidence surpasses the total number of cases reported over the prior 40 years [2,3].

Monkeypox is a zoonotic infection caused by an Orthopoxvirus endemic to Africa [4]. Historically, MPXV has typically spread via animal-to-human transmission [5]. However, during the 2022 outbreak, MPXV was primarily transmitted via skin-to-skin contact, especially in the men having sex with men (MSM) community [5]. This rapid human-to-human transmission is also being attributed to a possible new strain [6]. Monkeypox virus’ neurotropic potential has been suggested in animal models; previous studies have been done in prairie dog models investigating the best treatment for pain [7-9]. Studies have also shown that the MPXV virus induces T cell receptor-mediated T cell activation resulting in a potent inflammatory cascade [10]. Orthopoxviruses like MPXV modulate Nuclear Factor Kappa-Light-Chain (NFΚB) activation causing an inflammatory reaction, and Non-Steroidal Anti-Inflammatory Drugs (NSAIDs) have been shown to inhibit the nuclear factor NFKB, thereby being effective in pain control for patients with MPXV [11,12].

Monkeypox traditionally reveals symptoms 4-21 days after exposure [5]. Prodromal phase symptoms typically include headaches, back pain, myalgia, lymphadenopathy, and fatigue that last up to 4 weeks [13,14]. Skin eruptions and rashes subsequently develop on the face, hands, feet or in the anogenital region [15]. These lesions are often extremely painful until they crust over within 7-14 days of the rash onset [15]. Due to severe pain in some patients presenting with MPXV rash, the Centers for Disease Control (CDC) has urged providers to conduct further studies addressing the issue of pain management in MPXV patients [16]. Given the urgency of the need to disseminate information relevant to pain in MPXV, we have conducted a rapid review using a standardized methodology to identify studies reporting pain in patients infected with MPXV [17,18]. To the best of our knowledge, this is the first paper to review the pain characteristics and treatments for MPXV patients, as well as provide recommendations for an institutional framework to administer pain care for MPXV patients.

## Review

Methods

Data Source and Search Strategies

We conducted a rapid literature search that adheres to the reported guidelines of the Preferred Reporting Items for Systematic Reviews and Meta-Analyses (PRISMA) statement. After registering the literature search protocol on Open Science Framework (10.17605/OSF.IO/379MW), a comprehensive search of several databases from each database's inception to August 19, 2022, English language, was conducted. The databases included Ovid MEDLINE(R) and Epub Ahead of Print, In-Process & Other Non-Indexed Citations, and Daily, Ovid EMBASE, Ovid Cochrane Central Register of Controlled Trials, Ovid Cochrane Database of Systematic Reviews, and Scopus. The search strategy was designed and conducted by an experienced librarian (LP) with input from the study's principal investigator. Controlled vocabulary supplemented with keywords was used to search for pain associated with MPXV infection in humans. References were uploaded to Covidence for peer review screening.

The actual strategy employed, which lists all search terms used and how they were combined, is available in the Appendices.

Study Selection and Data Extraction

Two reviewers (AHC and CP) independently performed abstract/title screening searching for eligibility criteria. Manuscripts that fulfilled the criteria were reviewed by senior investigators. Any conflicts were resolved by the senior investigator (SG). Those meeting criteria for inclusion underwent data extraction. Data extraction was performed by research staff which included author and year of publication, type of study, number of participants, patient demographics, and any information regarding pain characteristics, physical exam, pain intensity, associated pain, and treatments. We included all published reports which presented a description of acute pain in patients diagnosed with MPXV virus infection. Acute pain was defined as pain that developed during the active infection. Review articles that did not include original clinical data were excluded.

Integration of Results

Relevant clinical information obtained during data extraction was synthesized by research staff and reviewed by senior investigators (CH, MH, JE, and SG). As described by protocol, the Newcastle-Ottawa Scale (NOS) was considered for assessing the quality of nonrandomized studies. However, due to the novelty of the current outbreak, manuscripts reporting pain symptoms were limited to case reports, case series, and retrospective studies that did not compare or study response to pain as the primary outcome. The quality analysis could not be conducted in all studies due to the lack of studies assessing pain as a primary or secondary outcome. Quality assessments were conducted on case series and case reports using the tool proposed by Murad, et al [19]. Data were analyzed using qualitative techniques given the paucity of large studies, heterogeneity of the data, and the lack of consistent reporting of pain outcome measures.

Ethical Considerations

The present study was only based on data collected from the published literature and was exempt from IRB review.

Results

**Figure 1:**
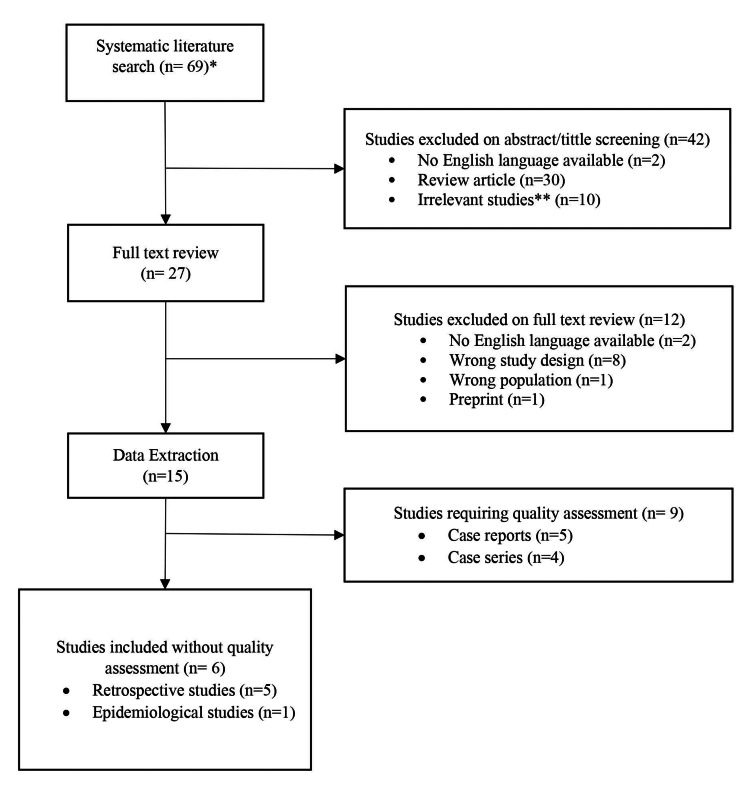
Manuscript selection description *No duplicates were found **Animal studies, other Poxvirus described, vaccines information, immuno-studies of monkeypox virus

Sixty-nine studies were identified in the initial search. After screening, 27 papers were considered for full-text review, and 15 manuscripts reporting acute pain from MPXV infection were ultimately included for data extraction. A complete description of the manuscript selection process is available in Figure 1.

Of the 15 included papers, five (33.33%) were case reports, four (26.6%) were case series, five (33.3%) were retrospective studies, and one (6.6%) was an epidemiological study (Figure 1). The full data extraction is described in Tables 1-3. Quality assessments of case series and case reports are reported in Table 4.

**Table 1:** Summary of included case reports N = number of patients Polymerase chain reaction or Reverse transcription-polymerase chain reaction was used in all case reports to diagnose monkeypox infection.

Author, Year	N	Age	Gender	Pain Initial Symptom?	Pain region	Pain Intensity	Treatment
Oprea, 2022 [20]	1	30	Male	Yes	Ano-rectal	Severe. No score reported	Topical treatment (not specified)
Costello, 2022 [21]	1	28	Male	Yes	Forehead	Not reported	Not reported
Hammerschlag, 2022 [22]	1	30s	Male	No	Genital	Not reported	Oral analgesia (not specified)
Pembi, 2022 [23]	1	30	Male	No	Lymphadenopathy	Not reported	Acetaminophen
Patrocinio-Jesus, 2022 [24]	1	31	Male	No	Lymphadenopathy	Not reported	Not reported

**Table 2:** Summary of included case series N = number of MPXV-positive patients, NR = not reported; PCR = polymerase chain reaction; MPXV = monkeypox virus

Author, Year	N	Mean Age	Gender, % male	Pain prevalence	Pain Initial Symptom?	Pain region	Pain Intensity	Method of diagnosis	Treatment
Pfafflin, 2022 [25]	6	NR	100%	Anal pain (n=6)	No	Anorectal	5/10 10/10 9/10 10/10 10/10 10/10	PCR	Metamizole 500 mg q6h Lidocaine 50 mg/g topical Stool softeners Ibuprofen
Patel, 2022 [15]	197	38	100%	Rectal pain (n=71) Sore throat (n=33) Penile oedema (n=31)	Yes	Right thigh Right sided neck pain Penis Ano-rectal	Severe. No score reported	PCR	Fentanyl Acetaminophen Ibuprofen Opioids Lidocaine gel Rectal suppositories Oral laxatives
Moschese, 2022 [26]	34	NR	100%	Patients hospitalized due to pain (n=1)	Yes	Not reported	Severe. No score reported	PCR	Analgesic therapy. Not specified
Thornhil, 2022 [4]	528	38	98%	Headache (n=145) Myalgia (n=165) Proctitis (n=75)	Not specified	Anorectal (21/528)	Severe. No score reported	PCR	No reported

**Table 3:** Summary of included retrospective and epidemiological studies N = number of MPXV-positive patients, NR = not reported; PCR = polymerase chain reaction

Author, Year	N	Mean Age	Gender, % male	Pain prevalence	Pain Initial Symptom?	Pain Region	Pain Intensity	Method of diagnosis	Treatment
Ogoina, 2022 [27]	40	37	77.5%	Not reported	Yes	NR	NR	Clinical judgement of the attending physician	NR
Girometti, 2022 [28]	54	41	100%	Inpatient treatment for severe pain (n=5)	No	Disseminated presentation	Severe. No score reported	PCR	Analgesia. Not described
De Baetselier, 2022 [29]	4	NR	100%	Painful vesicular rash (n=1)	Yes	Perianal rash	NR	PCR	NR
Huhn, 2005 [30]	34	26	53%	Headache (65%) Myalgias (56%)	No	Mouth sores	NR	Viral culture PCR Electron microscopy Immuno-histochemistry	NR
Croft, 2007 [31]	19	28	76%	Headache (n=13) Joint pain (n=6)	NR	NR	NR	PCR Immuno-histochemistry Virus culture Electron microscopy	NR
Yinka-Ogunleye, 2019 [32]	122	29	69%	Headache (n=61) Myalgia (n=42) Sore throat (n=45)	No	NR	NR	PCR IgM	NR

**Table 4:** Quality assessment Case Reports and Case series Quality assessment based on Murad, et al [19]. + = Not reported domain, ++ = Incomplete domain, +++ = Well documented domain

	Case Reports				
Authors	Selection	Ascertainment	Causality	Reporting	Total
Oprea, 2022 [20]	+	++	+++	++	++
Costello, 2022 [21]	+	++	+++	++	++
Hammerschlag, 2022 [22]	+	++	+++	++	++
Pembi, 2022 [23]	+	++	+++	++	++
Patrocinio-Jesus, 2022 [24]	+	++	+++	+	++
	Case Series				
Pfafflin, 2022 [25]	++	+++	+++	++	++
Patel, 2022 [15]	+++	+++	+++	++	++
Moschese, 2022 [26]	+++	+++	+++	+++	+++
Thornhil, 2022 [4]	++	+++	+++	+	++

A total of 1043 positive cases were included in this review. Patients included in this review were reported from case series (n=765), followed by retrospective and epidemiological studies (n=273) and case reports (n=5). Active MPXV infection in these patients was confirmed by polymerase-chain-reaction (PCR), virus culture, electron microscopy, and immunoglobulin M (IgM). Of these 1043 patients, 718 (57%) reported some type of pain.

Acute pain was mainly reported in case series (n=529, 68.7%), followed by retrospective/epidemiological studies (n=173, 63.4%), and case reports (n=5, 100%). Most subjects were male, and the major sources of pain described were musculoskeletal (n=261) and headaches (n=145). Genito-anal pain was specifically reported in 186 patients (case reports = 2, case series = 183, and retrospective/epidemiological studies = 1).

Rash is a cardinal symptom of MPXV infection, but only 10 papers described the association and intensity of pain associated with the vesicular rash.

Among patients who reported acute pain during the active infection, 77% (n=552) required outpatient treatment, and 23% (n=166) required inpatient treatment (case reports = 4, case series = 151, and retrospective/epidemiological studies = 11). Even though the reason for hospitalization was not described by most authors, in total, 38 patients were reported as requiring hospitalization due to rectal pain associated with anogenital lesions. Other reported reasons for hospitalization included secondary infection, sepsis, and quarantine precautions. All studies reported a complete recovery with no residual pain. Treatment options proposed by the authors include acetaminophen, ibuprofen, opioids, lidocaine gel, and metamizole. Specific mention was made of rectal suppositories containing emollients or the use of steroids with oral laxatives for cases involving severe anal pain.

Discussion

Summary of Evidence

Our team provides the first evidence synthesis to inform providers regarding pain management in their response to the MPXV outbreak. In most cases, the symptomatology was reported as mild and self-limited requiring outpatient treatment and isolation, but a considerable number of patients were admitted due to serious complications. A common morbidity was severe pain, and this was often the symptom prompting patients to seek medical attention. Pain control was a common reason for hospital admission [15,20,21,26,27,29]. Unfortunately, the severity and characteristics of pain were often not fully documented by the authors; however, the pain described in other Orthopoxviruses such as smallpox and chickenpox typically ranges from moderate to severe in intensity and is characterized by a ‘shooting’ quality [33].

Commonly associated manifestations included systemic signs and symptoms of fever, headaches, myalgias, mouth sores, and a disseminated skin rash with pustular or ulcerated lesions. A less common presentation, related to close physical contact, has also been reported as a local skin rash in the genital or anorectal region [34]. Genital and anorectal lesions are the main concern in the current outbreak as all patients reported with this local distribution have developed severe pain requiring medical attention [15,20,25]. Moreover, pustular lesions could appear before other systemic manifestations [15,20,21,26,27,29].

Severe pain persisting after acute disease or rash has been reported in other poxvirus infections. We were unable to find reports of chronic post-infection neuralgia in the current outbreak [35]. The pain associated with MPXV seems to be self-limited and resolves after the infection subsides [36] (Tables 1-3). The identified studies did not report the typical time course for pain resolution after infection. Interestingly, dysphagia has been identified as a predictor of disease severity [30].

Current literature supports pain management as the primary consideration for the current MPXV outbreak as more than half of the patients have reported varying degrees of pain. A rapid review can be helpful in circumstances such as a pandemic or new disease outbreak to help increase awareness and guide clinical decision-making, although the disadvantage is that controlled studies, large retrospective, or prospective reviews are not complete.

Currently, there is not enough information to standardize a pain treatment plan given the paucity of research in this area to date and the lack of randomized controlled trials. In fact, the treatment plan was reported only in case reports and case series. In cases of mild pain, outpatient treatments were a viable option utilizing over-the-counter medications. Treatment options such as acetaminophen, ibuprofen, and metamizole as well as topical treatment such as lidocaine gel were recommended in various articles in our review [15,23,25]​​​​​. The use of topical steroids and anesthetics has also been recommended by the CDC. In cases of severe anorectal pain, warm sitz baths, rectal suppositories containing emollients or steroids and oral laxatives should also be considered [15,20,22-26]. A single author reported the need for fentanyl and opioids for refractory pain. However, we suspect that refractory pain may be underreported due to the limited literature available, and a considerable number of patients will report refractory pain. The character of pain described is often neuropathic in nature, with a burning character at the site of the lesions, with the worst pain accompanying lesions near the anogenital region similar to other poxvirus infections [37,38]. For these reasons, the CDC has recommended the use of gabapentin and opioids for refractory pain related to MPXV based on anecdotal reports [39]. The benefit of tricyclic antidepressants has not been reported in the current outbreak. The benefit of opioids for pain control should be balanced with the risk of constipation, which could worsen the already severe proctitis reported by patients [39]. Even though patients with limited access to health care could benefit from home remedies, there is limited information to validate their beneficial effects.

Limitations

Due to the limited information on the current monkeypox outbreak, the present rapid review has several limitations. First, there was considerable methodologic heterogeneity among the identified studies. Relevant information regarding treatment and management considerations came from observational studies such as case reports and case series. Quantitative data such as epidemiological and retrospective studies provided valuable information regarding pain prevalence but did not report proposed treatment plans or pain descriptions in their cohorts. This could be explained as the pain experienced by patients has been underestimated during the current outbreak and highlighted the importance of the present rapid review.

Second, given that our goal was to publish a rapid review to disseminate information during a disease outbreak, we included only studies available in the English language, potentially limiting the data available to assess pain associated with MPXV. Moreover, to consolidate specific information regarding pain characteristics and management, our search strategy could have led to a selection bias. Thus, the pain prevalence reported in this study could be overestimated. Even though we were aware of this limitation, the objective of this review was not to merge epidemiological data but to provide a stepping stone for future studies to standardize treatment plans for severe pain during acute monkeypox infection. The data abstracted met this project objective, demonstrating the limited information currently available and the need for methodologically stronger projects that focused on this unmet need.

A third limitation is the significant clinical heterogeneity across patients. The limited evidence of high-quality studies and different methodological designs on the studies selected in this project precluded statistical analysis as patients reported by authors had different ages, lesions, and geographical locations. Due to this level of heterogeneity and the subjective nature of symptomology, outcome measures were not pooled.

Future direction

Providing pain care during outbreaks like MPXV, especially during the overlapping COVID-19 pandemic, can be extremely challenging and institutions and physicians need support to provide safe and effective pain care to their patients. To achieve this goal, future randomized controlled trials and comparative studies need to be conducted to help provide data for the best treatment for pain relief in this patient population. Timely dissemination of information on best practices to treat MPXV is critical to support the needs of patients in an evolving crisis.

## Conclusions

The current literature supports that pain management is a key consideration in the current MPXV outbreak as more than half of patients reported some degree of pain. Moreover, pain has been reported as one of the primary reasons for which patients with MPXV seek medical attention. There is no high-quality evidence to guide clinical decision-making at this time, but providers should be aware of reports of severe pain, particularly when the disease involves the anorectal region, and anticipate the need for commonly used adjuvants, anti-inflammatory medications, acetaminophen, topical therapies, and use of opioids in severe or refractory pain.

We recommend further investigation with prospective studies and encourage authors to report pain characteristics and treatment in their studies. Reports regarding persistent pain after infection would be helpful to further characterize long-term morbidity in patients with MPXV infection. Guidelines may be needed to help guide the best management to avoid morbidity in patients, particularly as adjuvants may play a key role but are not commonly utilized in published reports.
